# Eales’ disease: epidemiology, diagnostic and therapeutic concepts

**DOI:** 10.1186/s40942-021-00354-0

**Published:** 2022-01-04

**Authors:** Sergio Murillo López, Silvia Medina Medina, Fernando Murillo López

**Affiliations:** 1 Clínica del Ojo, Jaime Mendoza St. # H- 22. Torre Grazia 1st. Floor, La Paz, Bolivia; 2 Instituto Privado de Oftalmología, La Paz, Bolivia

**Keywords:** Eales’ disease, Occlusive retinal vasculopathy, Vitreous hemorrhage, Macular edema

## Abstract

**Background:**

To describe the epidemiological traits, clinical characteristics, diagnostic procedures, therapeutic interventions and evolution in a large series of patients with diagnosis of Eales’ disease.

**Methods:**

A clinical retrospective review of patients with Eales’ disease, evaluated and treated between April 2009 and April 2018, with a 1-year minimum follow-up. Thirty patients (59 eyes), were included. Age, sex, laboratory results (CBC, glycemia, protein electrophoresis, ACE levels) immunological profile and a Quantiferon-TB Gold Plus test were recorded. The patients were divided into groups according to their evolution, medical or surgical treatment, and visual outcomes.

**Results:**

Seventeen male patients and 13 female patients were included, and their ages ranged from 14 to 35 years. The Quantiferon-TB Gold Plus test was positive in 25 patients. Twenty-eight patients had unilateral vitreous hemorrhage, 10 of whom presented with vasculitis and non-perfusion areas in the contralateral eye, 9 presented contralateral peripheral neovascularization and 9 had contralateral fibrovascular proliferation. The remaining 2 patients presented with a rhegmatogenous retinal detachment. In 6 patients, conservative treatment with intravitreal anti-VEGF injections and photocoagulation was performed after the hemorrhage cleared. Twenty-two patients, required vitrectomy, with good visual outcomes. Macular edema was found in 16 eyes, which responded to periocular and/or systemic corticosteroid therapy, except for 9 eyes that required intravitreal bevacizumab, with complete resolution in 7 eyes and partial resolution in 2 eyes.

**Conclusions:**

Eales’ disease is a pathology of significant prevalence in our country. The distribution according to sex, tends to be equivalent. The etiology, even when it is not specifically determined, according to laboratory tests, confirms the probable immunologic response in the presence of *Mycobacterium tuberculosis* antigens. This is still a diagnosis of exclusion, and therefore, it is advisable to perform a complete laboratory work-up in each case. Timely application of laser and other medical treatments, help to avoid progression to more advanced stages and their complications. The surgical treatment of vitrectomy for vitreous hemorrhage, and/or tractional vitreous detachment yields good primary anatomical and functional outcomes. Secondary macular edema responds to periocular and intravitreal corticosteroids, and in refractory cases, the use of anti-VEGF therapy leads to an effective resolution.

## Background

Eales’ disease, constitutes an idiopathic occlusive retinal vasculopathy apparently multifactorial, and with a likely immunologic origin, which mainly affects the peripheral retina bilaterally in young patients predominantly males [1, 2]. The largest series of cases have been described in India and Southeast Asia [3]. In our country, we consider it to be fairly prevalent and relevant in a global context.

Its occlusive, inflammatory, vascular and potentially ischemic nature—generating extensive areas of non-perfusion and resulting neovascularization, as well as changes in the structure of the vitreous and its interaction with the retina, are responsible for its wide range of clinical manifestations depending on the evolution of the process. In its evolution we can find vitreous hemorrhages, macular edema, tractional retinal detachments, and less frequently, secondary rhegmatogenous or mixed RD, as well as secondary neovascular glaucoma.

Different therapeutic interventions, including pharmacological therapy, laser and/or surgery have been reported, and they are used independently or in combination according to the patient’s needs, and determined by the specific clinical situation.

In this study, we report epidemiological features, diagnostic procedures and therapeutic interventions with their results and evolution, in a large series of patients with a long follow-up.

## Patients and methods

A total of 30 patients with a diagnosis of Eales’ disease were included in this study. They were treated at the Retina and Vitreous Department at the Clinica del Ojo in La Paz, Bolivia, between April 2009 and April 2018.

Epidemiological data were collected, including age and sex, previous relevant systemic history and ophthalmological disease, primary reason for attending the clinic, clinical features, including best-corrected visual acuity, intraocular pressure and biomicroscopy of anterior and posterior segments. The results from diagnostic procedures included the following: fluorescein angiography, ocular ultrasound (when necessary) and optical coherence tomography of the macula.

Based on the clinical findings and the complementary diagnostic studies, we assigned a clinical stage for each patient according to the proposed classification of Saxena and Kumar [4] and the presence or absence of macular edema was also documented. Similarly, a laboratory work-up was obtained including: complete blood count, glycemia, protein electrophoresis, levels of angiotensin-converting enzyme (ACE), immunologic profile (PCR, sed rate, rheumatoid factor, ANA, antiDNA, antiCCP, cANCA, pANCA), Quantiferon-TB Gold Plus test, and chest X-ray.

Medical or surgical treatment of each eye was recorded, as well as the anatomic (clearing the visual media, retinal reattachment, RNV involution) and functional results (baseline best-corrected visual acuities and the ones obtained in the last follow-up).

Patients with insufficient data in the clinical chart and those who did not complete at least 1 year of follow-up were excluded from the study.

## Results

Thirty patients (60 eyes) were diagnosed with Eales’ disease between 2009 and 2018, 53% of those were females and 47% were male. The median age of presentation was 34 years, with a range from 16 to 49, and a greater prevalence was found between the second and fourth decades of life (Table 1).

**Table 1:** Epidemiological data and relevant systemic previous history

Sex	
Female	53%
Male	47%
Age	
< 20 years old	6.7%
21–30 years old	30.0%
31–40 years old	33.3%
41–50 years old	30.0%
Previous medical history	
None	93.4%
Systemic hypertension	3.3%
Rheumatoid arthritis	3.3%

Systemic arterial hypertension was detected in 1 patient and quiescent rheumatoid arthritis was detected in 1 patient. There was no relevant systemic past history in the remaining patients (Table 1), and no patient had a previous relevant ophthalmological history.

The main reason for attending the clinic was unilateral sudden painless loss of vision (80%), followed by myodesopsias. One patient was found during a routine examination (Table 2).

**Table 2:** Reason for initial consultation and clinical findings

Reason for consultation	
Diminished VA	80.0%
Myodesopsias	16.7%
Asymptomatic	3.3%
Findings in the symptomatic eye	
VH	27 eyes
RRD + Fibrovascular proliferation	1 eye
RRD + peripheral sheathing	1 eye
VH and mixed RD	1 eye
Findings in the contralateral eye	
Peripheral vascular sheathing and non-perfusion areas	11 eyes
Peripheral RNV*	9 eyes
Fibrovascular proliferation areas	7 eyes
Absolute neovascular glaucoma	3 eyes

Visual Acuity (VA), Vitreous Hemorrhage (VH), Reghmatogenous Retinal Detachment (RRD), Retinal Detachment (RD), Retinal Neovascularization (RNV)

The best-corrected visual acuity of the eye responsible for the consultation, ranged between HM and 20/40, and the contralateral eye’s vision ranged between 20/50 and 20/20. Fifty-nine eyes did not show any abnormalities in biomicroscopy of the anterior segment. Only one eye showed inflammatory signs (cells 2 + and fine keratic precipitates). Intraocular pressures were found to be within normal limits in all cases. The most common clinical finding of the posterior segment was unilateral vitreous hemorrhage of variable degree (Table 2). This occurred in 27 eyes, varying from grade II-III, allowing good visualization of the peripheral retina in several cases. One patient presented vitreous hemorrhage associated with a mixed retinal detachment and the remaining 2 patients presented with peripheral sheathing and fibrous proliferation.

**Fig. 1:**
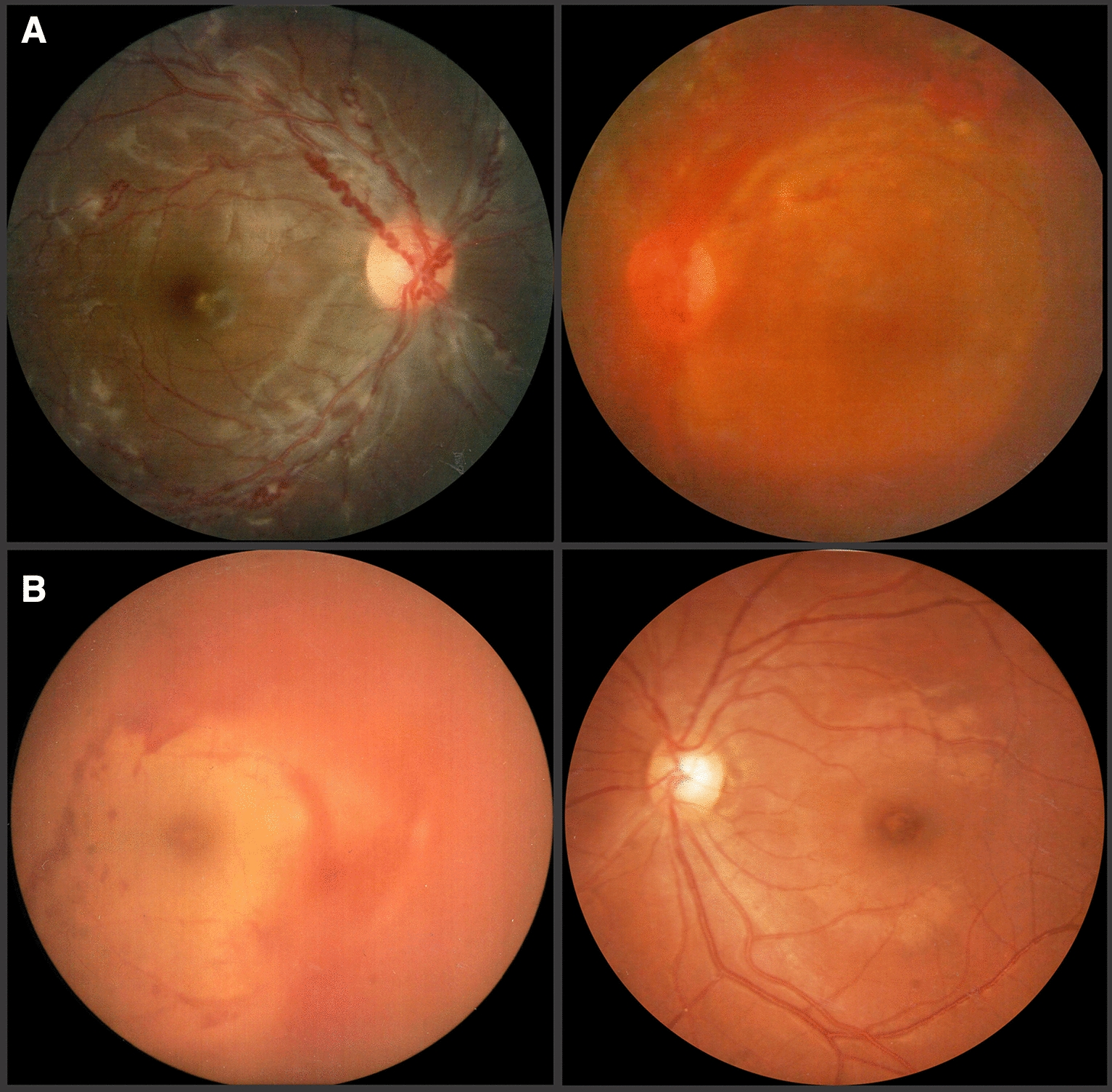
**A** Retinal fundus photography showing vascular tortuosity and sheathing in the right eye and vitreous hemorrhage in the left, in a 19 year-old male, with active Eales’ disease. **B** Retinography of a 23 year-old female with vitreous hemorrhage on the right eye and an apparent normal fundus in the left

**Fig. 2:**
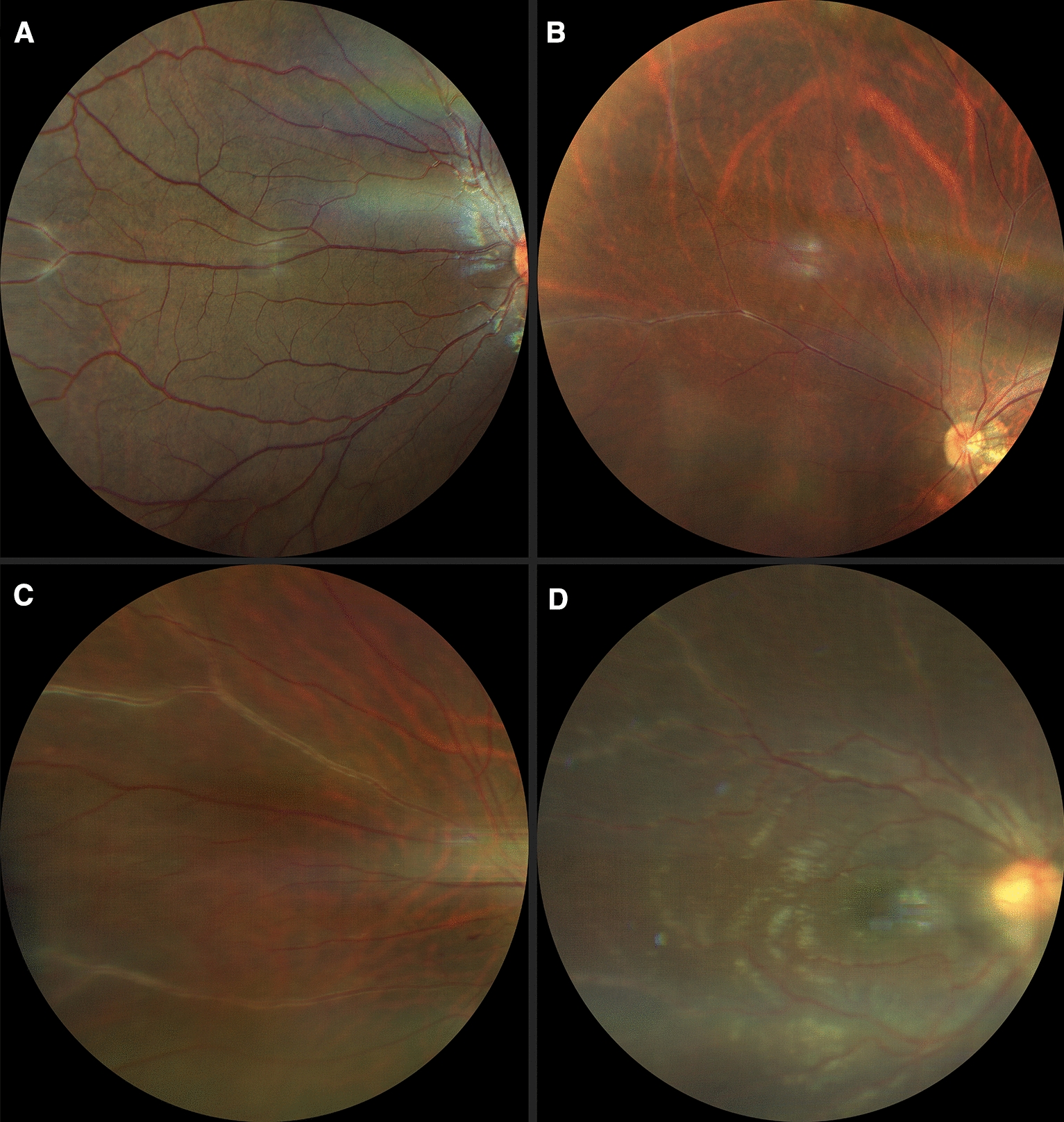
Fundus pictures showing vascular sheathing to different degrees: **A**, **B** mild, **C** moderate and **D** severe

In 11 eyes of the 30 patients, there was retinal vascular sheathing, predominantly in the mid-periphery, compromising the posterior pole in 36.4% of cases and areas of non-perfusion in the contralateral eye (Figs. 1, 2). In 9 patients, there was contralateral peripheral neovascularization, 3 patients presented absolute neovascular glaucoma, and in the remaining 7 patients areas of fibrovascular proliferation in the contralateral eye, were clinically appreciated (Table 2).

A clinical stage was assigned for each eye based on the classification proposed by Saxena and Kumar [1]. Most of our patients (45%) were in a stage IIIb in the symptomatic eye and IIa (18%) in the contralateral eye (Table 3).

**Table 3:** Eales’ disease staging according to Saxena and Kumar (Global percentage and comparative table showing the asymmetric presentation in each patient)

STAGE (Global)	
I	3.3%
IIa	18.3%
IIb	15.0%
IIIa	11.7%
IIIb	45.0%
IVa	1.7%
IVb	5.0%

STAGE (Comparative)		
Patient	Right Eye	Left Eye
1	IIIb	IIa
2	IIIa	IIa
3	IIIa	IIb
4	IIIb	IIb
5	I	IIIb
6	IIIb	IVb
7	IVa	IIa
8	IIIb	IVb
9	IIb	IIIb
10	IIb	IIIb
11	IIIb	IIa
12	IIb	IIIb
13	IIIb	IIIa
14	IIIb	IIa
15	IIIa	IIIb
16	IIIb	IIb
17	IIIb	IIa
18	IIa	IIIb
19	IIIb	IIb
20	IIIb	IIa
21	IIb	IIIb
22	IIIa	IIIb
23	IIIb	IIa
24	IIIb	IIIa
25	IIIb	I
26	IIa	IIIb
27	IIIb	IIa
28	IVb	IIIb
29	IIIa	IIIb
30	IIIb	IIb

**Fig. 3:**
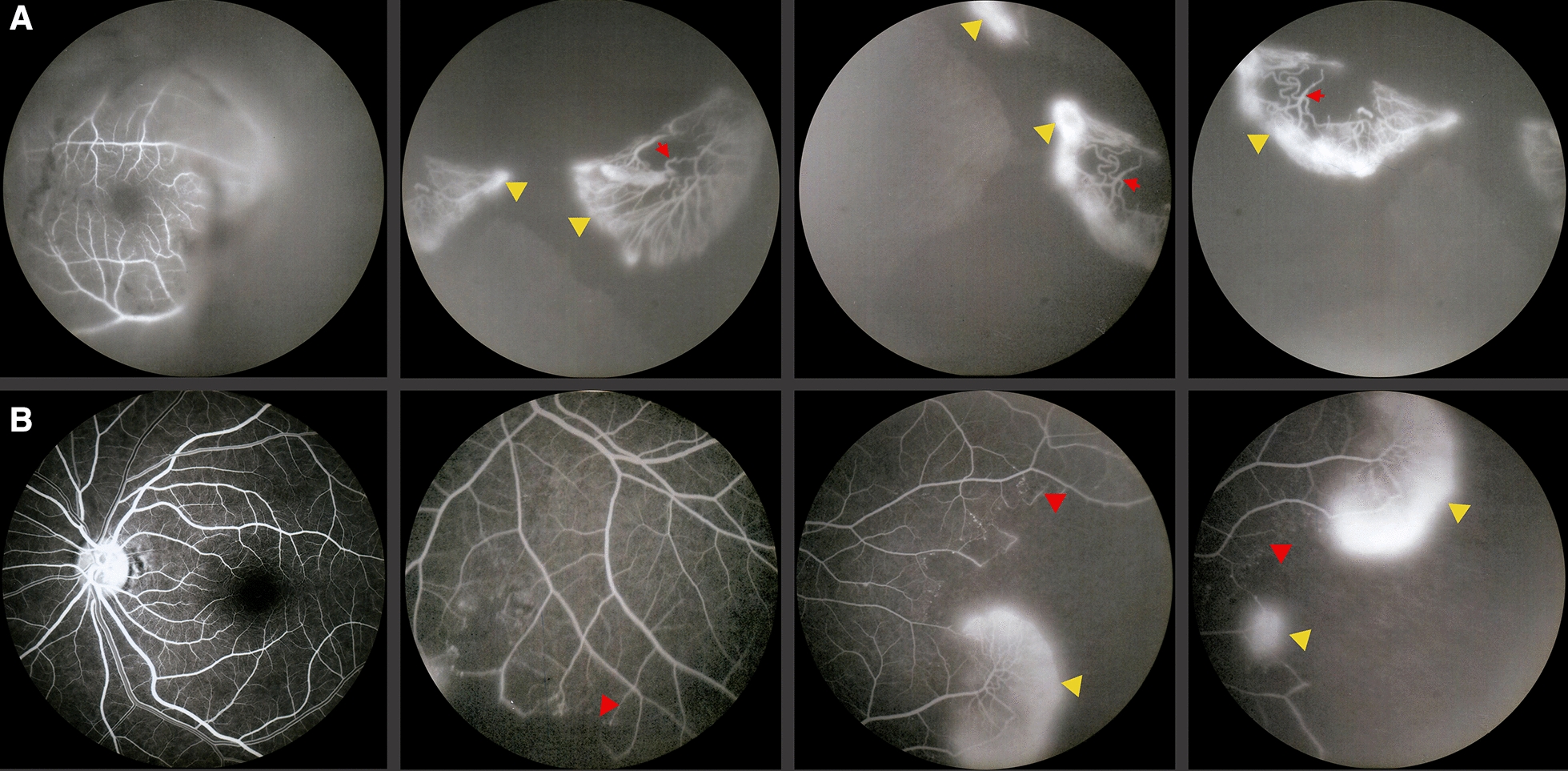
Fluorescein angiography from the same patient as in Fig. 1B. Blocked fluorescence due to vitreous hemorrage in the right eye (**A**) and peripheral vascular anomalies: AV shunts (red arrows) with hyperfluorescent leakage corroborating neovascularization elsewhere (yellow arrowheads) in both eyes. It also shows peripheral hypofluorescence in non-perfusion ischemic areas (red arrowheads) in the left eye (**B**)

**Fig. 4:**
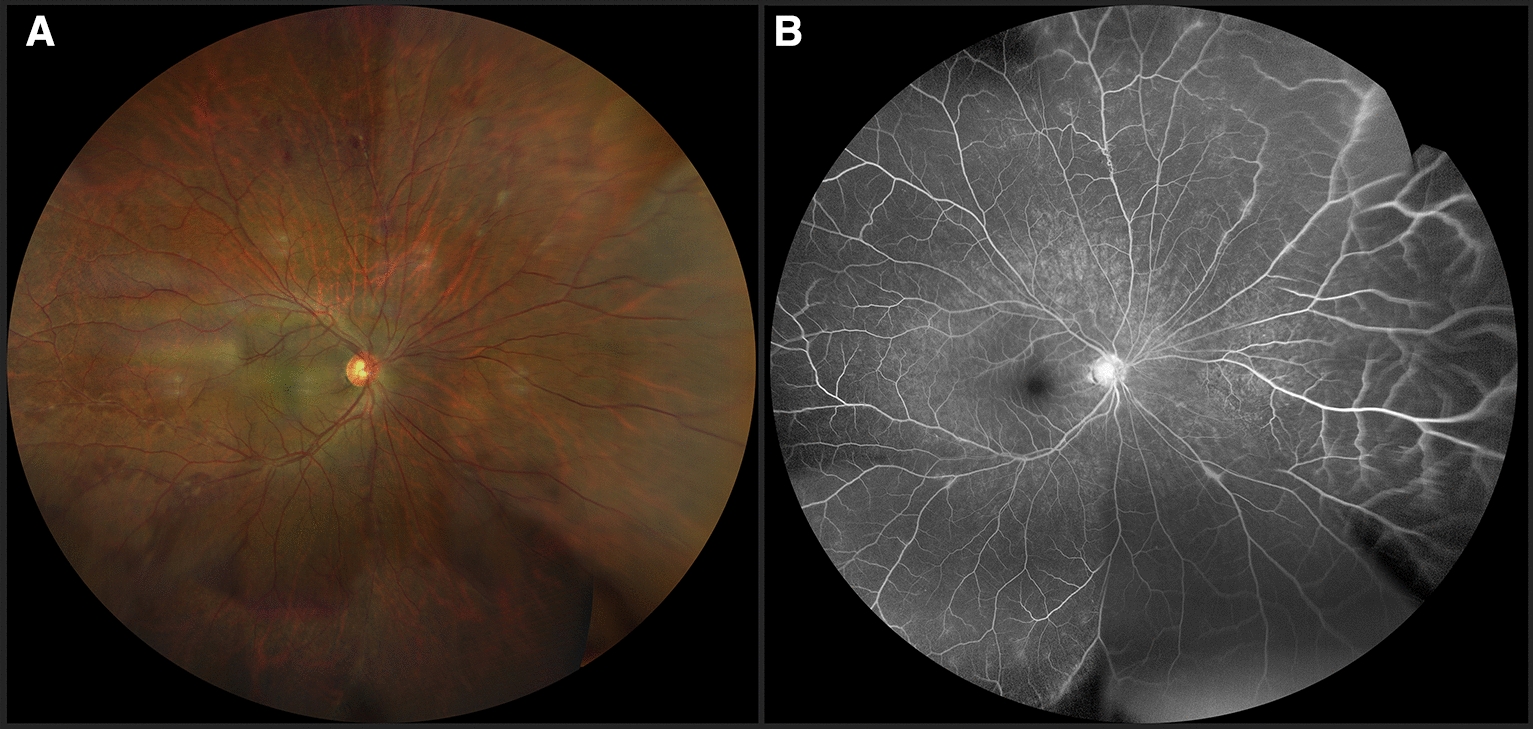
**A** Composed widefield retinography showing peripheral vascular sheathing, peripheral hemorrhages, and apparent nasal non-perfusion areas. **B** Peripheral nasal non-perfusion areas and vascular tortuosity and sheathing confirmed on composed widefield retinal fluorescein angiography in the same eye

FA showed areas of non-perfusion with a predominant location posterior to the equator in the mid-periphery (Figs. 3, 4) and RNV with leakage in the late phases of 50.8% (eyes that we were able to visualize), followed by areas of non-perfusion in 28.8%, and peripheral sheathing with vascular changes (tortuosity, changes in the vascular caliber, AV shunts) in the contralateral remaining eyes (5%) (Table 4). Cystoid macular edema was evidenced by FA in 10 eyes, with late diffuse leakage in 7 cases and a petaloid pattern in the remaining 3 (Table 4).

**Table 4:** Findings in complementary ocular studies

FA findings	
Non-perfusion areas and RNV	50.0%
Non-perfusion areas	28.3%
Discrete vascular changes (Vascular sheathing)	5.0%
Diffuse macular edema	16.7% (10 eyes)
Not able to evaluate	15.0%
Ocular ultrasound	
Intact Retino-choroidal complex	66.7% (6 eyes)
Reduced Axial Length and tissue disorganization	33.3% (3 eyes)
Macular OCT	
Macular Edema	27% (16 eyes)

In the eyes with dense vitreous hemorrhage (9 eyes) an ultrasound was performed, which showed integrity of the retinochoroidal complex in all cases. All eyes were examined with Optical Coherence Tomography (OCT) of the macula at the time of diagnosis or after clearing of the media. Macular edema was present in 16 of the eyes (27%), with central thickening and intraretinal cysts in 14 of the cases, 3 of which showed evidence of vitreofoveal traction, and the remaining 2 eyes showed diffuse perifoveal thickening (Table 4).

Twenty-three patients (76.7%) had a positive Quantiferon-TB Gold Plus test, and the remaining laboratory results were all within normal limits.

**Fig. 5:**
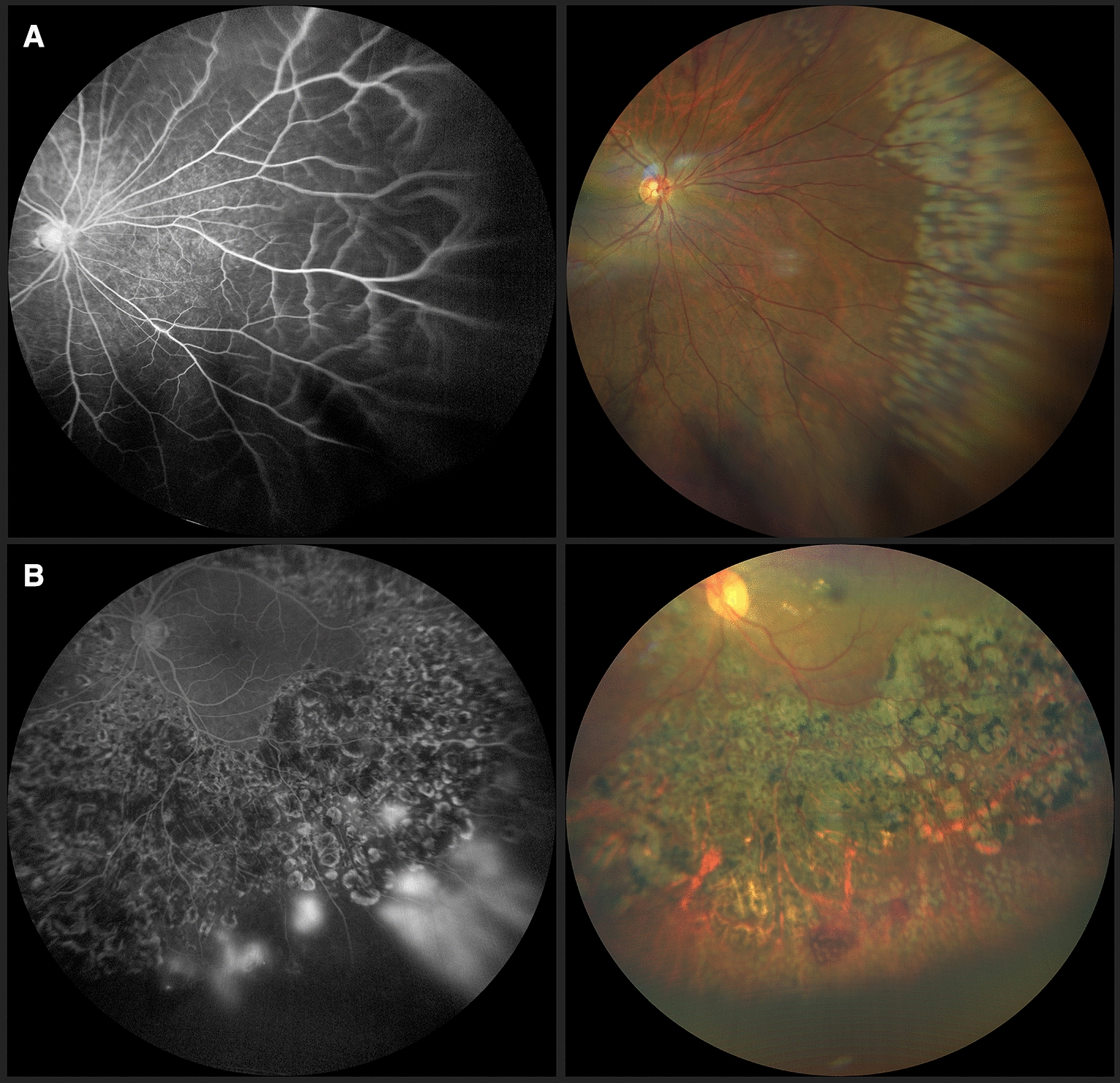
**A** Widefield fluorangiography showing nasal peripheral non-perfusion areas (left), Retinography immediately post laser application of non-perfusion areas guided by fluorangiography (right) **B** Late phase of fluorescein angiography shows peripheral leakage confirming neovascularization in a previously laser treated patient (left). Fundus photography with recent laser spots on the identified leakage and non-perfusion areas (right)

Regarding treatment, conservative management was followed in eyes without vitreous hemorrhage, in those patients with vitreous hemorrhage grade I or at least 4 weeks of evolution, and in those eyes whose media cleared in less than 8 weeks, with intravitreal anti-VEGF according to the evolution and retinal photocoagulation in areas of non-perfusion after clearing of the media (59%), guided by fluorescein angiography, once the effect of the anti-VEGF wore off (Fig. 5).

**Fig. 6:**
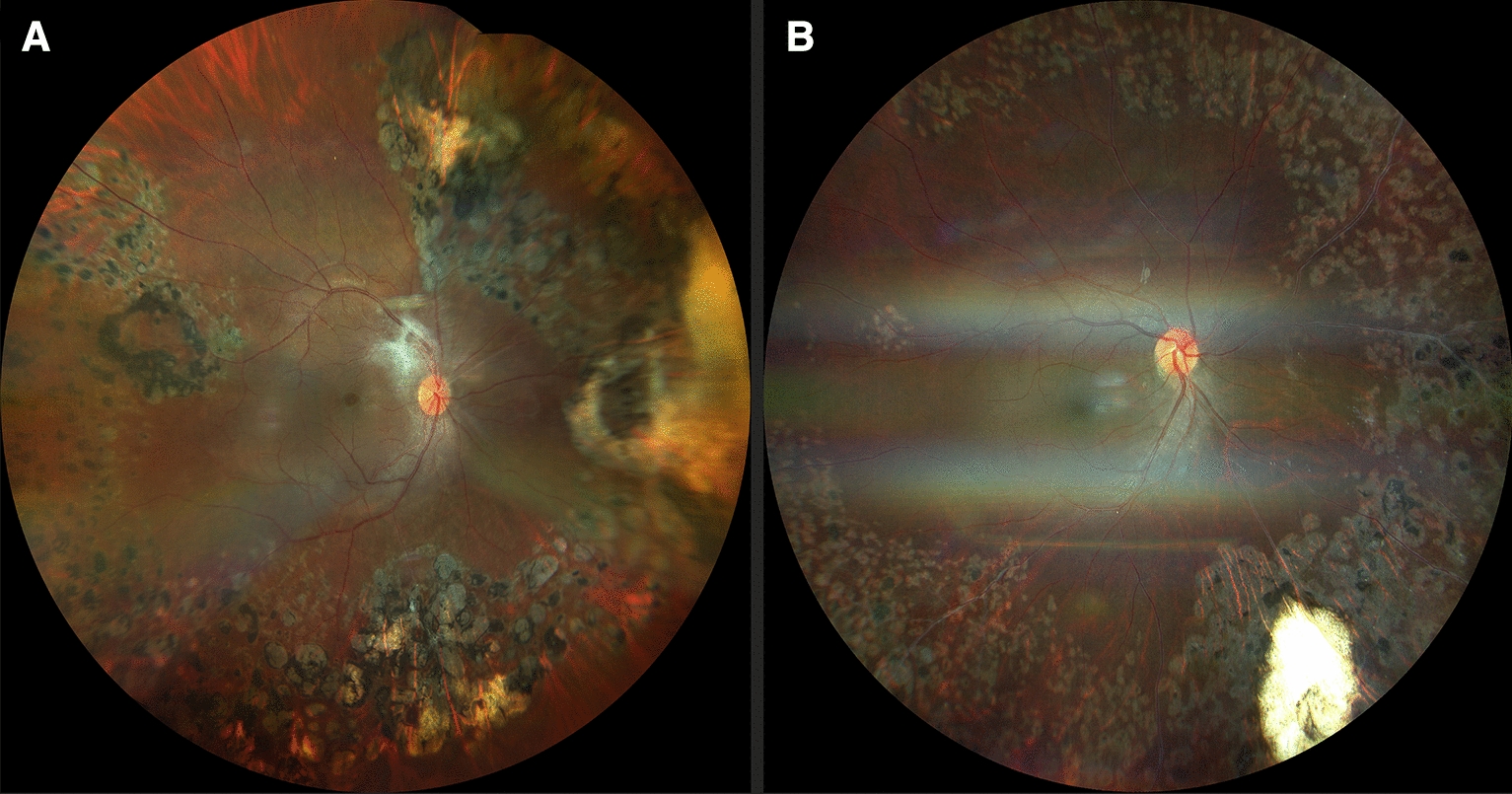
**A** Postvitrectomy retinography of a female patient, with a history of vitreous hemorrhage in the right eye. Laser scars distributed on neovascularization and non-perfusion areas identified during surgery, and residual fibrosis on the superotemporal vascular arcade are shown. **B** Postoperative fundus photography of a 17-year-old female with a history of vitreous hemorrhage. Image shows persistent vascular sheathing in nasal and inferotemporal retina and laser scars

**Fig. 7:**
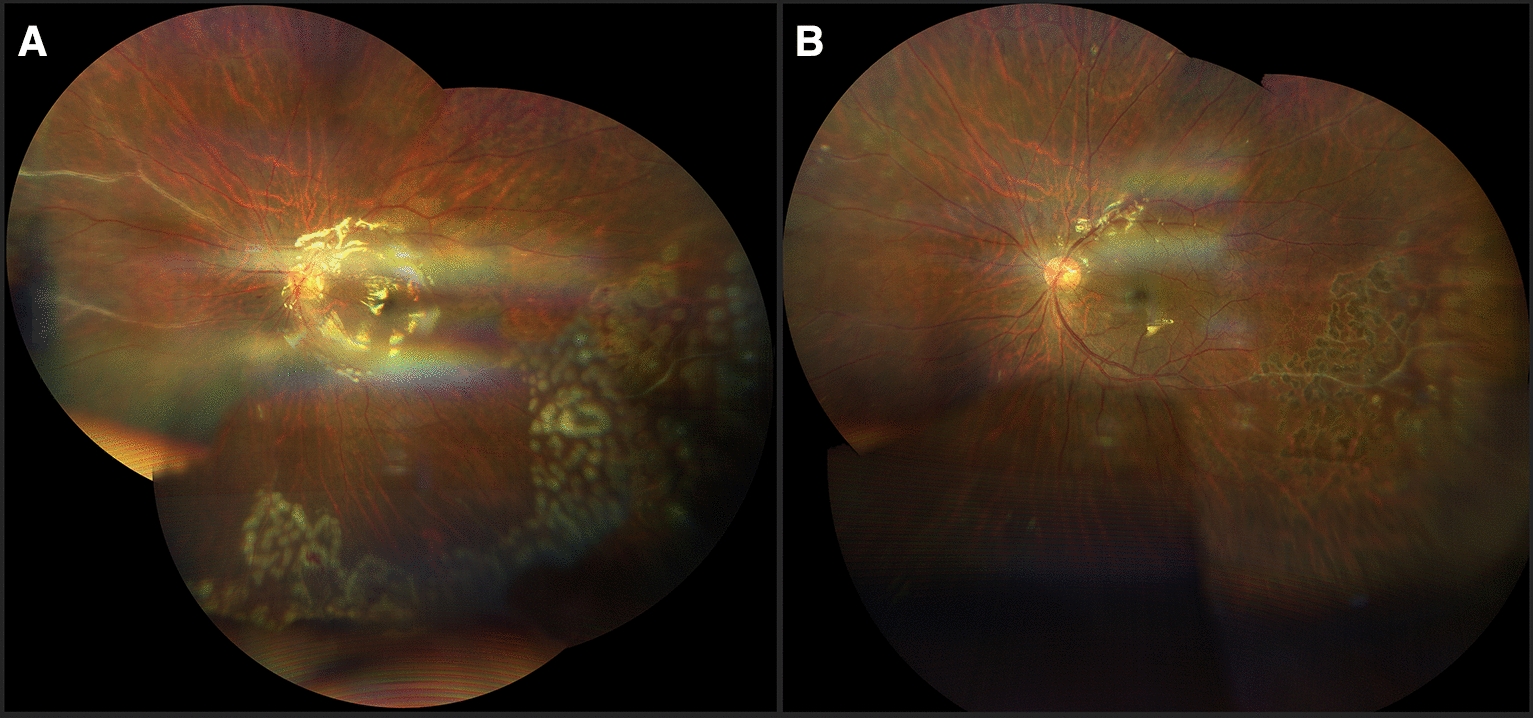
**A** Two weeks postoperative male, with a history of vitreous hemorrhage. Vitrectomy, with silicon oil tamponade and photocoagulation of ischemic areas was performed, and retinography showed persistent nasal vascular sheathing and recent laser spots on non-perfusion areas, identified during surgery. **B** Six weeks postoperatively fundus images of the same patient showed improvement of peripheral nasal vascular sheathing and laser scars in the inferotemporal quadrants

In 20 eyes with vitreous hemorrhage where there was no spontaneous clearing after anti-VEGF and/or more than 2 months of evolution without spontaneous clearing of the hemorrhage (34%) a pars plana vitrectomy was performed with laser endophotocoagulation (Figs. 6, 7), and associated with a scleral buckle and posterior retinal reattachment in those cases with mixed peripheral retinal detachment. In 18 eyes, clearing of the media was achieved and regression of the retinal neovascularization was confirmed by control angiography. The two remaining cases required additional posterior photocoagulation post-vitrectomy. All patients received periocular corticosteroids during the active inflammatory phase of the disease and in 38% of cases treatment with systemic corticosteroids (prednisone PO 1 mg/kg for 7 days with a gradual reduction of the dose depending on the individual response of each patient) (Table 5).

**Table 5:** Summary of treatments performed in each eye

Treatment	
Periocular corticosteroids	100%
Systemic corticosteroids	38%
Laser photocoagulation	95% (33% + Vitrectomy)
Vitrectomy + Laser endophotocoagulation	33.3%
FA guided Photocoagulation + anti VEGF	28.3%
FA guided photocoagulation	30.0%
Scleral buckle + Vitrectomy + Laser endophotocoagulation	1.7%
Scleral buckle + Cryoretinopexy + Post-op photocoagulation	1.7%
No treatment (NLP eyes)	5.0%
Macular Edema Management	
Periocular Corticosteroids	43.8%
AntiVEGF	56.2% Bevacizumab 43.7% Aflibercept 12.5%
Intravitreal dexamethasone implant (Ozurdex)	Ozurdex 6.2%

Patients with retinal detachment, were treated with scleral buckle + cryoretinopexy and scleral buckle + pars plana vitrectomy, both with effective reattachment of the retina and visual recovery (Table 5).

**Fig. 8:**
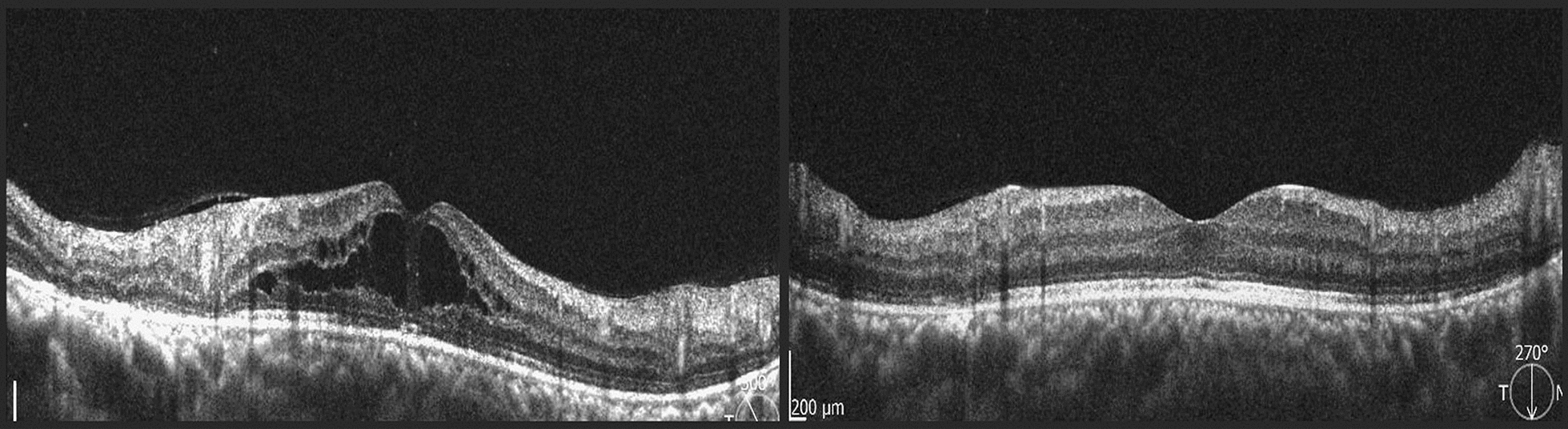
Macular optical coherence tomography of a 15-year-old patient at initial presentation (left) with cystoid macular edema, and post-treatment with periocular steroids (right)

With regard to the patients with macular edema, 7 eyes received treatment with periocular and/or systemic corticosteroids, with edema resolution, flattening of the retinal thickening and improvement of visual acuity in all cases, without complications (Fig. 7). The remaining 9 eyes received intravitreal bevacizumab, with complete resolution in 7 eyes and partial resolution in 2. All refractory cases received aflibercept and one of them received intravitreal placement of dexamethasone implant (Ozurdex) with significant edema reduction, but no complete resolution (Table 5). All these eyes, improved their visual acuity to different degrees following treatment (Fig. 8).

In 100% of cases clearing of the media was achieved, as well as regression of the neovascularization and retinal reattachment (in the corresponding cases). All patients presented functional improvement (except those who had a stage IVb at the time of initial diagnosis) with favorable changes or stability in visual acuity (Table 6).

**Table 6:** Functional Results, Comparison of initial and last follow-up Best-Corrected Visual Acuity

	Initial BCVA	Final BCVA
NLP	3 eyes	3 eyes
HM to 20/400	12 eyes	1 eye
20/200 to 20/80	11 eyes	6 eyes
20/70 to 20/50	9 eyes	10 eyes
20/40 to 20/20	25 eyes	40 eyes

## Discussion

The largest series of cases with Eales’ disease reported in the literature come from India [2–4], Southeast Asia [4] and Germany [5]. The epidemiology of this disease in Latin America has not been reported. In our country, however, it constitutes a pathology of significant frequency, comparable with that of other countries. This epidemiologic finding becomes relevant considering the significant prevalence in populations of different racial origins, suggesting that-genetic makeup does not determine the etiology-, or the development of the disease, and reinforcing instead the most accepted theory that links the origin of this vasculopathy with an abnormal immunologic response to the antigenic fragments of *Mycobacterium tuberculosis* [2, 3, 6]. This finding also highlights the fact that in Bolivia, we still register a high incidence and prevalence of tuberculosis [7].

Immunology-based etiopathogenesis has been proposed, as a reaction to genetic material and the presence of non-viable *Mycobacterium tuberculosis* fractions, which has been supported by strong evidence. Furthermore, statistically significant portions of M. tuberculosis DNA have been detected upon vitreous PCR analysis, and in epirretinal membrane analysis form Eales’ disease patients. Moreover, other retrospective and prospective studies also found other Mycobacterium (chelonae and fortuitum) [6] using optimized seminested polymerase chain reactions, reinforcing their causal relationship and extending in to other Mycobacterium species, in addition to tuberculosis. In contrast, some authors performed RT-PCR on vitreous biopsies and found a significant Mycobacteria inoculum in at least 50% of their samples; reinforcing the possibility of vasculitis being a reaction to an active infectious agent and not just an immune response [8].

On the other hand, a possible association with autoimmune systemic disease has been reported, and neurological and hematological disorders have also been mentioned [2]. Our series showed coexisting autoimmune disease in only one patient, but no other clinical or paraclinical analysis suggested other systemic diseases.

Immunohistochemical analysis showed lymphocytic infiltration, especially T lymphocytes, consistent with type IV hypersensitivity, which is a characteristic response to tuberculin, but can also be an expression of the autoimmune mechanism triggered by retinal S-antigen [9].

Vitreous biochemical analysis showed significant increase in several growth factors, specially VEGF, in contrast to a PEDF reduction –a potent ischemia inhibitor [10]- and increased reactive substances, such as TBARS, and decreased E and C vitamins, superoxide dismutase, glutathione and glutathione peroxidase, suggesting oxidative stress [11].

Our case series demonstrates that this disease is most common in young adults and the sex distribution is equivalent, which differs from the classic descriptions of a clear male gender dominance [1, 2, 5, 12], but it matches smaller case series reflecting a more even gender distribution [13].

A total of 76.6% of patients in our study had a positive Quantiferon-TB Gold Plus test, which is of relative value, since it only demonstrates immunologic memory of Mycobacteria. Since tuberculosis is endemic in our country, our population received the BCG vaccine, which reduces the specificity of this test for the detection of active tuberculosis cases [8].

We noticed bilateral involvement in 100% of our cases. However, it is important to mention the asymmetric evolution of a significant percentage of these cases, which allowed us to intervene at the early stages of many contralateral eyes in those patients with unilateral symptoms. This fact, also reinforces our recommendation of a careful evaluation (under pharmacological dilation) looking for subtle or peripheral signs in the periphery of asymptomatic eyes.

Only one case in our series, presented with signs of anterior uveitis, primarily cell and fine keratic precipitates. In fact, anterior segment inflammation is not usually part of these patients’ presentation and in those few cases where it was present it may have been due to spillover phenomenon [12], in those cases with a severe stage IIIb inflammatory process.

The most common reason for initial consultation was a reduction in visual acuity, secondary to vitreous hemorrhage, in patients previously asymptomatic and without relevant ocular or systemic comorbidity. The clinical stages associated with vitreous hemorrhage correlated to those with retinal neovascularization, confirming the cause-effect relationship. However, despite the advanced stages of these patients, the evolution is favorable if treated in a timely manner, because it lacks other components that could exacerbate the process, such as those of diabetes. Additionally, even though they share similar clinical and evolutionary characteristics, the behavior of Eales’ disease is less aggressive than that of proliferative diabetic retinopathy. We found this phenomenon even in cases with large areas of non-perfusion and/or aggressive neovascularization, probably because Eales’ pathology does not originate from a systemic metabolic disorder, or permanent endothelial dysfunction such as diabetic retinopathy [10].

Retinal vasculitis is located in the mid periphery, post-equatorial or equatorial in 64% of cases with a central predominant component in 36%. Some cases in early stages with close follow-up of its progression or recurrence post-treatment, display new spots of vasculitis in different meridians, always in the mid-periphery or closer to the center, but not in the most peripheral sectors. However, areas of vascular shutdown, extend to the pre-equatorial and extreme periphery in some cases where neovascularization tends to develop in transition areas between perfused and ischemic retina, which is why careful clinical exploration and complementary studies, especially fluorescein angiography should be able to demonstrate the most peripheral locations up to 360°, with new wide-field techniques probably optimal for evaluation and follow-up.

Similarly, associated macular edema is less common than in other retinal pathologies, the treatment response to a timely intervention is favorable, and the results are stable over time. Spectral domain OCT is an ideal tool for the diagnosis and follow-up of macular edema as well as abnormalities in the vitreoretinal interphase that could develop. OCT-A could also be valuable to evaluate other associated phenomena, such as macular ischemia, that could easily explain visual loss in some cases [12, 14], with an added advantage of its non-invasive quality and reproducible results.

Even though the clinical characteristics of Eales’ are usually quite distinctive and match those of the series of cases, this disease remains an exclusion diagnosis, which must be considered on the basis of clinical findings since it lacks a specific, conclusive diagnostic test. Therefore, all laboratory tests and referrals to other specialties -if needed- to rule out other causes of retinal vasculitis must be carried out without delay.

There were some differences regarding clinical features in our series compared to others, 100% of our patients showed bilateral compromise, compared to 73 and 87% reported in the largest case series in Asia [2, 4] and Europe [5], however, during follow-up these numbers increased to 81 and 98% respectively, reinforcing that asymmetry is frequent and both eyes are involved at some point, responding to previously established etiopathogenic mechanisms. Also, recently developed diagnostic tools, such as ultra-wide-field angiography and retinography are able to detect this changes on early stages. Vascular features in our patients showed venous sheathing in most of the cases, associated to vascular dilatation and tortuosity, microhemorrhages, AV shunts and neovascularization in the transition areas, between perfused and ischemic retina, these changes were consistent with previous reports. However, our patients did not present telangiectasias or microaneurysms, which are the first manifestation of internal retinal-blood barrier disruption in other retinopathies, different from Eales’ disease.

As for topographic features in our series, the main difference is a significant number of centrally located clinical characteristics-up to 36%-compared to the 10% rate, stablished by previous reports [2–5]. However, we must point out, that most of our patients didn't show disc neovascularization, compared with variable rates-between 1 and 6.2%-in other studies [2, 4], which can be attributed to the fact, that even if central compromise is common, corresponding non-perfusion and ischemic areas weren’t equivalent in extension. Additionally, we noticed that cystoid macular edema is the most frequent form of macular affection. 27% of cases showed vitreofoveal traction component and just 2% of them had epiretinal membranes; similarly to other series outcomes.

Combined treatment with anti-VEGF, systemic, periocular and/or intraocular corticosteroids, or external or intraoperatory laser photocoagulation in ischemic areas depending on the case, has shown favorable results [12, 15, 16], with structural and functional improvement. Similarly, macular edema and abnormalities in the vitreoretinal interphase, although less frequent, have been demonstrated to be treatable with the expected visual improvement.

Ocular comorbidities, such as retinal detachment, respond quite well to conventional surgical treatments, and we found that inflammation does not play a determinant role in the postoperative evolution. Obviously, the appropriate peri and postoperative control of any sign of inflammation must be taken into consideration to minimize its impact, and following surgery, once the retina is reattached, it is important to complete the ablation treatment of ischemic areas if they are present. There is still a need to establish diagnostic and therapeutic protocols that best fit the clinical stage and individual characteristics of each case (including the contralateral eye’s condition, presence of macular edema, etc.). Additionally, we will surely find more efficient applications of current technology such as angiographic studies of ultra-wide field, new generation anti-VEGF, and sustained release intraocular steroid devices, which will improve our diagnostic and therapeutic capabilities and help achieve more favorable visual outcomes.

## Conclusions

Eales’ disease is an ocular pathology with significant prevalence in our country. This bilateral vasculitis shows a similar frequency in young patients of both sexes, with specific clinical findings, but no specific diagnostic test, which is why a full laboratory work-up must be obtained for these patients in all cases.

The initial symptom is often a vitreous hemorrhage, usually without a preceding ocular history, and because of the bilateral nature of Eales it mandates a thorough examination of the fellow eye. With an early diagnosis, initiating treatment is significantly easier, and the results of the contralateral eye are usually favorable.

Treatment of the underlying pathology, as well as any secondary ocular comorbidity has a good prognosis and the rational, combined use of antiangiogenic, corticosteroid and laser therapy may provide a synergistic effect. Surgery has formal indications, and its outcome is favorable when combined with adequate pharmacologic control of the underlying process to minimize its impact. Our study has limitations because of its retrospective nature; future investigations with a prospective design are required to establish adequate diagnostic and therapeutic protocols that include the latest technology to optimize visual outcomes.

## Data Availability

The datasets analyzed during the current study are available from the corresponding author on reasonable request.
